# Structural Insights Into the 5′UG/3′GU Wobble Tandem in Complex With Ba^2+^ Cation

**DOI:** 10.3389/fmolb.2021.762786

**Published:** 2022-01-13

**Authors:** Agnieszka Ruszkowska, Ya Ying Zheng, Song Mao, Milosz Ruszkowski, Jia Sheng

**Affiliations:** ^1^ Institute of Bioorganic Chemistry, Polish Academy of Sciences, Poznan, Poland; ^2^ Department of Chemistry, The RNA Institute, University at Albany, State University of New York, Albany, NY, United States

**Keywords:** GU wobble pair, X-ray crystallography, RNA, structure, metal ion

## Abstract

G•U wobble base pair frequently occurs in RNA structures. The unique chemical, thermodynamic, and structural properties of the G•U pair are widely exploited in RNA biology. In several RNA molecules, the G•U pair plays key roles in folding, ribozyme catalysis, and interactions with proteins. G•U may occur as a single pair or in tandem motifs with different geometries, electrostatics, and thermodynamics, further extending its biological functions. The metal binding affinity, which is essential for RNA folding, catalysis, and other interactions, differs with respect to the tandem motif type due to the different electrostatic potentials of the major grooves. In this work, we present the crystal structure of an RNA 8-mer duplex r[UCGUGCGA]_2_, providing detailed structural insights into the tandem motif I (5′UG/3′GU) complexed with Ba^2+^ cation. We compare the electrostatic potential of the presented motif I major groove with previously published structures of tandem motifs I, II (5′GU/3′UG), and III (5′GG/3′UU). A local patch of a strongly negative electrostatic potential in the major groove of the presented structure forms the metal binding site with the contributions of three oxygen atoms from the tandem. These results give us a better understanding of the G•U tandem motif I as a divalent metal binder, a feature essential for RNA functions.

## Introduction

Watson–Crick (WC) base pairs shape the RNA double-helical landscape. However, multiple non-WC interactions (Leontis et al., 2002) have been distinguished in RNA structures and implicated in various biological functions of RNA (Westhof and Fritsch, 2000; Chandrasekhar and Malathhi, 2003; Brown, 2020). The G•U wobble base pair is the most frequent among the non-WC base pairs in RNA molecules. Its existence was proposed by F. Crick over 50 years ago, alongside the hypothesis that G•U plays an important role in the decoding of messenger RNA (mRNA) codons (Crick., 1966). Since then, G•U pairs have been found in multiple classes of RNA, including messenger RNA (mRNA) (Benard et al., 1998), ribosomal RNA (rRNA) (Woese et al., 1983; Gutell et al., 1994), transfer RNA (tRNA) (Ladner et al., 1975), small nuclear RNA (Wu and Manley, 1992; Sashital et al., 2004; Sashital et al., 2007), and ribozymes [such as group I and II introns, and in hepatitis delta virus (HDV)] (Cech et al., 1981; Allain and Varani, 1995; Strobel and Cech, 1995; Been and Wickham, 1997; Nishikawa et al., 1997; Colmenarejo and Tinoco, 1999).

**FIGURE 1:**
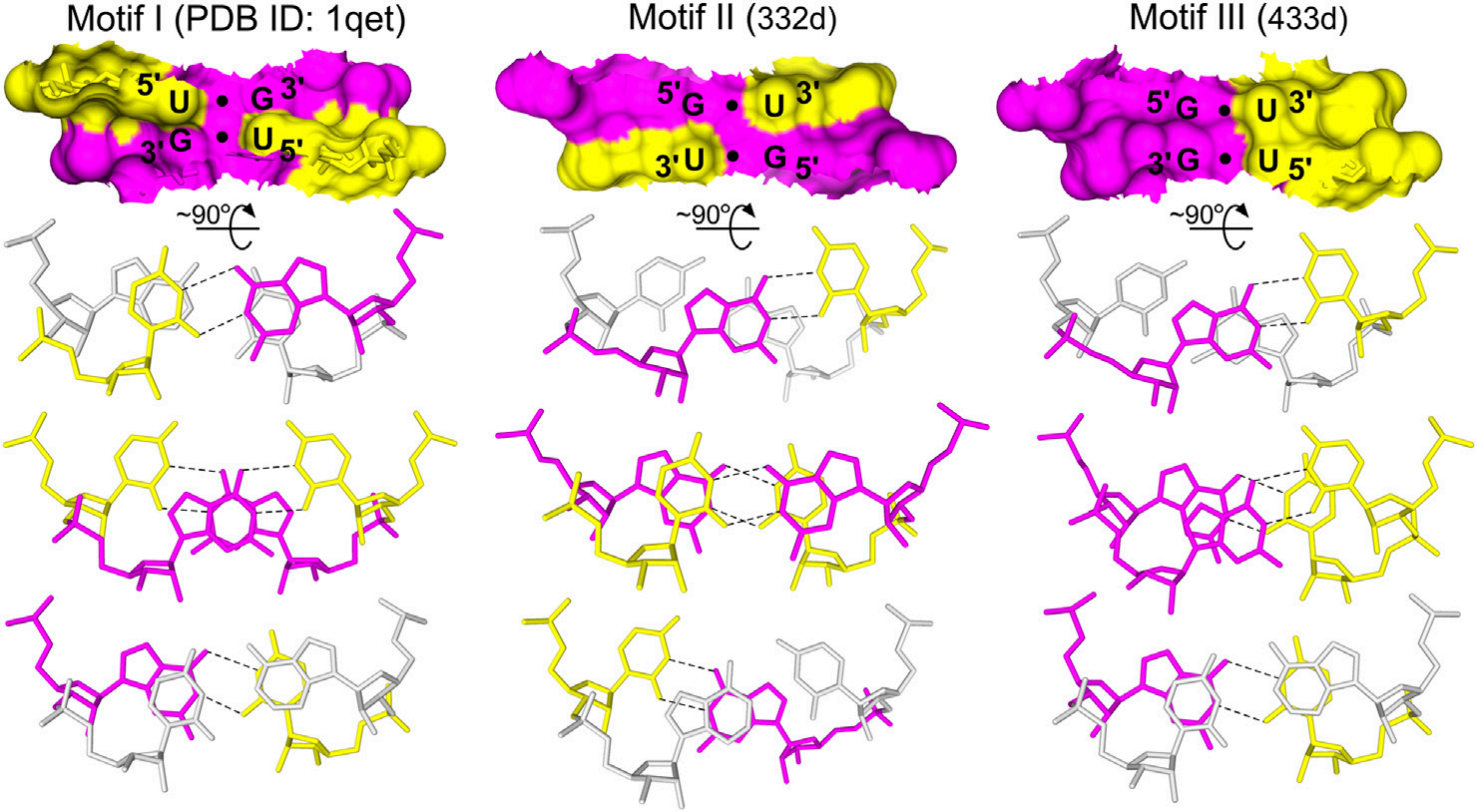
The three types of G•U tandem motifs. The tandem motifs are shown as surface representations based on the NMR and X-ray structures (Biswas and Sundaralingam, 1997; McDowell et al., 1997; Trikha et al., 1999). The stacking pattern characteristic for each motif is shown, including the base pairs flanking the G•U tandems (*gray*). In G•U tandems, G is *magenta* and U is *yellow*. Hydrogen bonds (*black dashed lines*) are presented only for G•U pairs.

The G•U pairs can be accommodated within A-form RNA helices with minimal structural distortions. In a typical G•U base pair, the uracil is displaced into the major groove of the RNA helix. However, other types of G•U pairing, i.e., the bifurcated G•U pairs (non-helical regions), the tautomeric G•U pairs (all positions of the codon/anticodon triplets), and the minor groove-shifted G•U pair (tRNA^Lys^ wobble position of the codon/anticodon triplet) have been observed (reviewed in Westhof et al., 2019). G•U pairs usually interact on their Watson–Crick edges, exploiting the subtleties of the G and U electronic configurations (reviewed in Westhof et al., 2019). Beyond numerous types of single G•U pairing, the regular G•U pair can also form tandems that are often present in rRNA structures (Gautheret et al., 1995). The types of G•U tandems vary in terms of their structural, electrostatic, and thermodynamic properties (Xu et al., 2007). The frequency of the different tandem motifs in rRNA follows the trend of 5′UG/3′GU (motif I) > 5′GG/3′UU (motif III) > 5′GU/3′UG (motif II) (Figure 1). The same trend applies to the thermodynamic stabilities of the G•U tandems (He et al., 1991; Gautheret et al., 1995; Wu et al., 1995; Deng and Sundaralingam, 2000).

The significance of the G•U base pair in RNA structures is emphasized by its high conservation (Sprinzl et al., 1996). Throughout the three domains of life, the G3•U70 pair is a major determinant of the amino acid acceptor identity of tRNA^Ala^ (Hou and Schimmel, 1988; McClain and Foss, 1988; Chong et al., 2018). The unique chemical groups of the G•U pair exposed to the major and minor grooves, its thermodynamic stability, conformational flexibility, and the non-isostericity of G•U compared to the WC pair are essential attributes for its biological functions (Varani and McClain, 2000; Ananth et al., 2013). The exocyclic N2 amino group of G exposed to the minor groove of G•U 1) mediates contacts with proteins, e.g., alanyl-tRNA synthetase (Musier-Forsyth et al., 1991; Ananth et al., 2013; Naganuma et al., 2014; Chong et al., 2018); 2) is involved in the 5′ splice site selection and stabilization of the transition state in group I self-splicing introns (Strobel and Cech, 1995; 1996); and 3) thermodynamically stabilizes interactions within the catalytic core of group II self-splicing introns (Boudvillain and Pyle, 1998; Konforti et al., 1998). The major groove of the G•U pair, due to its deep negative potential, may function as a recognition site for metals, amino acids such as R, H, K, and N, and other positively charged ligands (Varani and McClain, 2000; Chen et al., 2010; Ananth et al., 2013). The unbounded O4 carbonyl group of U is one of the G3•U70 tRNA^Ala^ elements recognized by alanyl-tRNA synthetase (Naganuma et al., 2014). The conserved G (G•U) of the catalytic triad in group II introns is involved in RNA triplex formation through its major groove edge (Keating et al., 2010; Manigrasso et al., 2020); the G chemical groups exposed to the major groove are critical for catalysis (Konforti et al., 1998). The non-isosteric nature of G•U, along with the presence of the N2 amino group in the G•U minor groove, is an important element for the *Tetrahymena* ribozyme catalytic activity (Strobel and Cech, 1995) and tRNA^Ala^ aminoacylation (Hou and Schimmel, 1988; Park et al., 1989; Ananth et al., 2013). Moreover, the non-isostericity of G•U plays a role in RNA tertiary interactions. The shift of U into the major groove of the G•U pair allows it to form a minor groove suitable for close contact with the WC pair of the other helix, creating the so-called along-groove packing motif (Gagnon and Steinberg, 2002; Ananth et al., 2013).

Here, we present the crystal structure of an RNA duplex containing motif I of the G•U wobble tandem. In the previous study, the major groove of G•U wobble motif I has been determined as less negative than the major groove of motifs II and III and accordingly suggested as the least favorable metal binding site among the G•U tandems (Xu et al., 2007). In this work, we observed that the 5′UG/3′GU tandem complexed with the Ba^2+^ cation, showing that G•U wobble motif I attracts metal ions and functions as their potent binding site. Structural analysis of the presented RNA duplex confirmed the A-form helix and indicated a few unique features of the G•U tandem.

## Results and Discussion

### Overall Structure Quality

The crystal of the r(UCGUGCGA)_2_ duplex was obtained even though an equimolar of its complementary strand UCGCACGA was used for crystallization as a hybrid duplex. The presence of a divalent cation (Ba^2+^), bound in the major groove (see below), might be the major driving force behind the formation of the r(UCGUGCGA)_2_ duplex. The crystals in the *H*32 space group, with one duplex in the asymmetric unit, diffracted X-rays to 2.2 Å resolution. The detailed diffraction data and model refinement statistics are listed in Table 1. There are 10 structures of RNA octanucleotides in the Protein Data Bank (PDB), determined at resolutions between 1.15 and 2.6 Å.

**TABLE 1:** Diffraction data and refinement statistics.

Data collection	
Wavelength (Å)	1.0000
Space group	*H*32
Unit cell parameters, *a* = *b*, *c* (Å)	44.0, 122.3
Resolution (Å)^a^	36.39-2.21 (2.35-2.21)
Unique reflections^a^	2,460 (386)
Multiplicity^a^	5.7 (5.5)
Completeness (%)^a^	99.5 (100.0)
*R*_merge_ (%)^a^	4.0 (112.3)
<*I*/σ(*I)*>^a^	17.3 (1.9)
CC_1/2_ (%)^a^	99.9 (81.0)
Refinement	
*R*_free_ reflections	123 (5%)
No. of atoms (non-H)	339
RNA	336
Ba^2+^	1
H_2_O	2
*R*_work_/*R*_free_ (%)	23.4/28.6
RMSD from ideal geometry	
Bond length (Å)	0.000
Bond angle (deg)	1.584
Average B, all atoms (Å^2^)	74
PDB ID	7ouo

a Values in parentheses correspond to the highest resolution shell.

**FIGURE 2:**
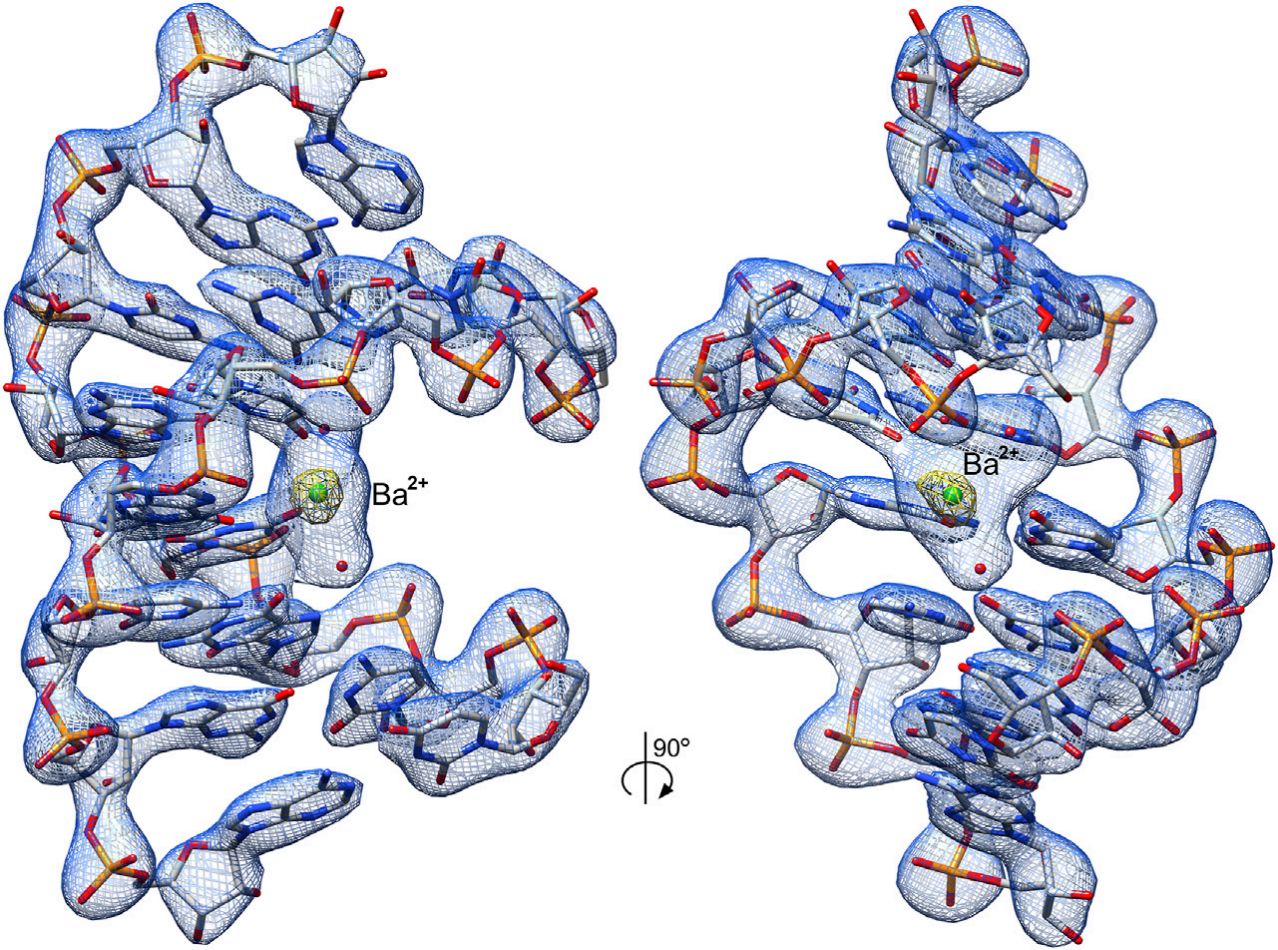
Crystal structure of the r(UCGUGCGA)_2_ duplex. The structure contains the 5′UG/3′GU wobble tandem constituting the binding site of Ba^2+^ (*green ball*) and two water molecules (*red balls*). The 2*F*_o_–*F*_c_ electron density map (*blue mesh*) around the RNA duplex is contoured at the 1.5σ level. The anomalous difference electron density map (*yellow mesh*) is shown at the 7σ level; the strong map peak verifies the presence of Ba^2+^.

**FIGURE 3:**
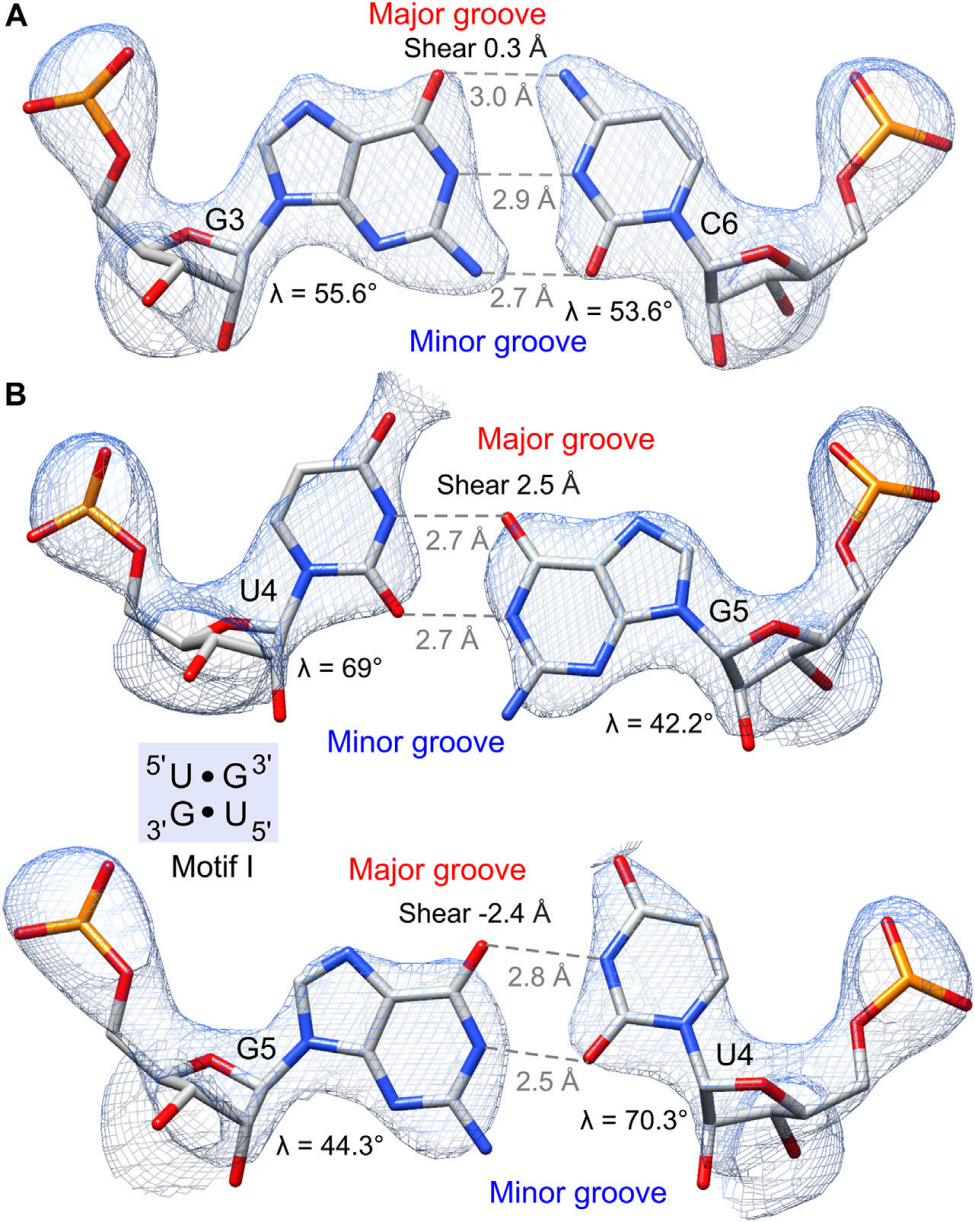
Hydrogen bonding pattern and geometry of the G•U pair and its canonical counterpart. The 2*F*_o_–*F*_c_ electron density map (*blue mesh*) of wobble pairs is contoured at the 1.5σ level. **(A)** G–C pair preceding the 5′UG/3′GU tandem. **(B)** 5′UG/3′GU tandem representing motif I (*light blue box*). The guanosine N7, guanosine O6, and the unpaired uridine O4 carbonyl group of G•U pairs are exposed to the major groove and generate a region of negative potential. The non-hydrogen-bonded N2 amino groups of guanosines are positioned in the minor groove. Unique structural features of G•U such as shear and unequal glycosidic bond angles (*λ*) at G and U are indicated.

The obtained electron density maps allowed tracing RNA unambiguously and modeling one Ba^2+^ cation and two water molecules in its coordination sphere (Figure 2). The strong peak (9.9σ level) in the anomalous difference electron density map corroborated the presence of Ba^2+^; at the used X-ray energy of 12,400 eV, the *f*′′ of barium is ∼4.1e. Individual base pairs were well-defined in the maps for canonical WC pairs (Figure 3A) and for the 5′UG/3′GU wobble tandem (Figure 3B).

### The Major Groove Created by the 5′UG/3′GU Wobble Tandem Is Optimal for Binding Divalent Cations

**FIGURE 4:**
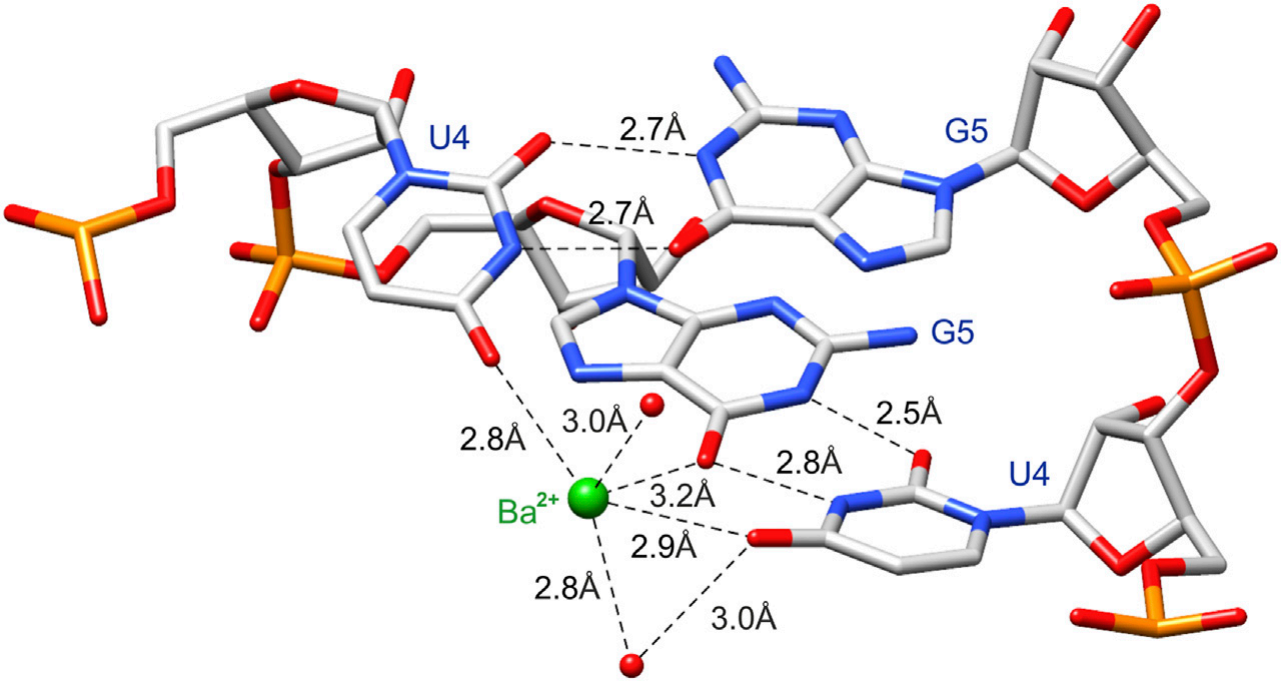
The cation binding site is created by functional groups of the 5′UG/3′GU wobble tandem [O6 carbonyl group of G5 (chain A) and O4 carbonyl group of U4 in both chains] poised into the major groove. Ba^2+^ and water molecules are marked as *green* and *red balls*, respectively.

Ba^2+^ was bound in the duplex major groove by the exo-O4 atoms of U4 residues in both chains and the O6 atom of G5 (chain A) (Figures 2 and 4). Two water molecules coordinated Ba^2+^ in the major groove. The coordination sphere of Ba^2+^ was incomplete, although seemingly compatible with the most common Ba^2+^ coordination number of 9. More ligands of the Ba^2+^ cation (e.g., four more water molecules) may not be visible in the electron density maps due to the limited data resolution.

**FIGURE 5:**
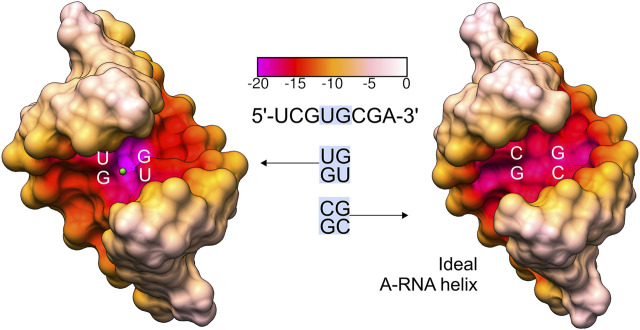
Comparison of the major groove electrostatic potential in the presented 5′UG/3′GU wobble tandem (motif I) and ideal A-helix RNA containing canonical 5′CG/3′GC counterparts (generated using *Coot* software) (Emsley et al., 2010). Ba^2+^ is shown as a *green ball*. The electrostatic surface was calculated using the APBS/PDB2PQR server (default settings) (Baker et al., 2001; Dolinsky et al., 2004; Jurrus et al., 2018).

The G•U wobble pair is negatively charged with the group composition of guanosine N7, guanosine O6, and the unpaired O4 carbonyl of uridine, which are exposed to the major groove forming a region of strong negative electrostatic potential (Figure 3B) (McDowell and Turner, 1996; Varani and McClain, 2000). A comparison between the surface electrostatic potential of the presented 5′UG/3′GU wobble tandem duplex and an ideal RNA helix containing the canonical 5′CG/3′GC motif showed clear centralization of the negative charge within the major groove of the wobble tandem (Figure 5). In contrast, WC pairs exposed their amino groups into the major groove and disturbed the negative electrostatic potential (Figures 3A and 5).

The negatively polarized surface within the major groove of the G•U wobble pair has a strong affinity for divalent metal ions (Ott et al., 1993; Allain and Varani, 1995; Konforti et al., 1998). This effect is even stronger for two consecutive G•U pairs (Cate and Doudna, 1996; McDowell and Turner, 1996; Kieft and Tinoco, 1997). In the presented structure, the Ba^2+^ cation was located in the center of the 5′UG/3′GU tandem (Figures 2 and 4). Universally, the G•U pair is polarized, and the G side is more negative than U. In the 5′UG/3′GU wobble tandem (motif I), the two Gs belonged to different strands, and the center of the tandem was the most negative part of the duplex (Figures 4 and 5) (Xu et al., 2007).

The database of the metal ion binding sites in RNA (MeRNA) indicates that the G•U pair major groove is the most common metal binding RNA motif (Stefan et al., 2006). Divalent metal ions are usually crucial for RNA structure and function. Metal ions can counteract the repulsion between the negatively charged phosphate backbone and stabilize various RNA tertiary structures for diverse functions (Cate and Doudna, 1996; Draper, 2004; Woodson, 2005; Marcia and Pyle, 2014). For example, the capacity of the G•U pair to interact with metal ions was postulated as an important factor for RNA catalysis, i.e., partially hydrated Mg^2+^ ion interacting with N7 and O6 of the reverse G•U pair in the HDV ribozyme active site directly participates in RNA cleavage reactions (Chen et al., 2010).

**FIGURE 6:**
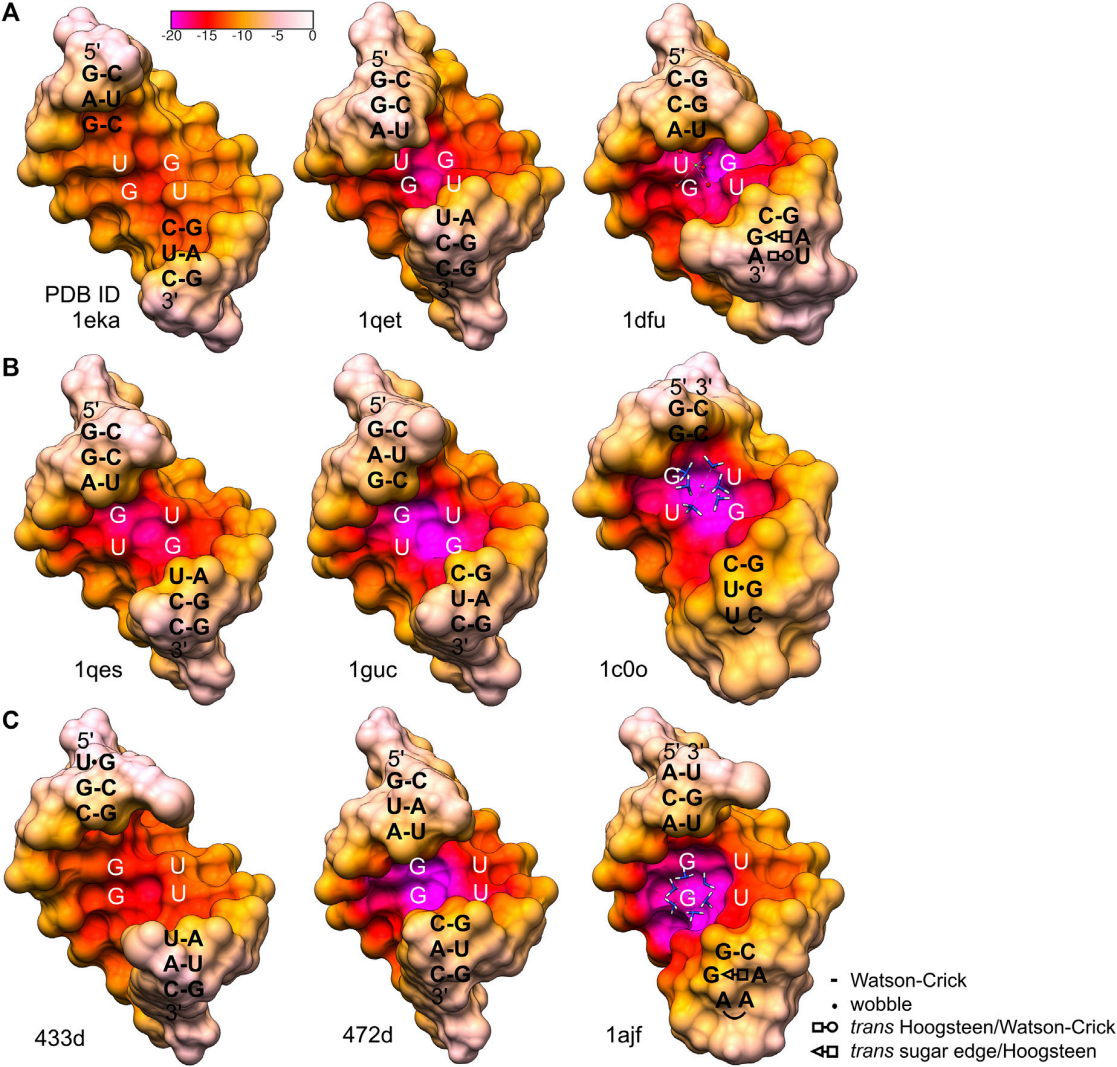
Electrostatic potential of the major grooves found in the X-ray and NMR structures containing different types of wobble tandems (McDowell and Turner, 1996; Kieft and Tinoco, 1997; McDowell et al., 1997; Colmenarejo and Tinoco, 1999; Trikha et al., 1999; Chen et al., 2000; Deng and Sundaralingam, 2000; Lu and Steitz, 2000). **(A)** 5′UG/3′GU wobble tandem, motif I. **(B)** 5′GU/3′UG wobble tandem, motif II. 1c0o shows the P5 hairpin of a group I intron complexed with [Co(NH3)_6_]^3+^. **(C)** 5′GU/3′GU wobble tandem, motif III. 1ajf is a model of a group I intron P5b stem–loop structure complexed with [Co(NH3)_6_]^3+^. The electrostatic surface was calculated by the APBS/PDB2PQR server using default settings (Baker et al., 2001; Dolinsky et al., 2004; Jurrus et al., 2018). The original structures 1dfu, 433d, and 1ajf were truncated to octamers to correspond to the other presented motifs. Interactions in sequences flanking the G•U tandem motifs are represented as shown in the legend, according to (Leontis et al., 2002).

Generally, G•U wobble pairs enhance the negative potential in the major groove. However, the stacking patterns and major groove sizes associated with various G•U tandem motifs affect the absolute electrostatic potential (Xu et al., 2007). The major groove created by a single G•U pair and the three motifs of the wobble tandem (I, II, and III) differed in widths and consequently showed diverse contributions of their phosphate backbones to electrostatic potential (formal electrostatic potential; Figure 6). The G•U tandem motif I (Figure 6A) has been proposed to exhibit less negative formal electrostatic potential than the G•U tandem of motifs II and III (Xu et al., 2007) (Figures 6B, C). The 1eka structure (Chen et al., 2000), representing the G•U tandem motif I contained a significantly extended major groove (Figure 6A); the inter-strand phosphate–phosphate distance for this helix was 19.8 Å (Xu et al., 2007). Its wide major groove caused a lower concentration of negative potential from the bases and the backbone. In effect, the negativity of the G•U pair was diffused and the major grove electrostatic potential was similar or even weaker than those of its canonical counterparts (Xu et al., 2007) (Figure 6). The major groove of the presented helix, also containing motif I wobble tandem, revealed a direct inter-strand phosphate–phosphate distance of 16.5 Å (Supplementary Table S1). Thus, the major groove width was closer to those of the G•U tandem motifs II and III (Xu et al., 2007) (Figures 5 and 6) and canonical counterparts (∼14.9 Å in an ideal A-helix; Supplementary Table S1). Moreover, analysis of the surface electrostatic potential of this duplex indicated the presence of a negatively charged region within the major groove, which was, in fact, the Ba^2+^ cation binding site (Figures 4 and 5).

Another G•U tandem motif I, found in loop E of 5S rRNA [1dfu (Lu and Steitz, 2000), truncated to the duplex of octamers], was characterized by quite negative formal electrostatic potential of the major groove that binds Mg^2+^ (Figure 6A). The direct inter-strand phosphate–phosphate distance within the G•U tandem in that structure was ∼15.4 Å. The general geometry of the helix in the 1dfu structure was affected by other non-WC base pairs in the neighborhood of the 5′UG/3′GU tandem (1dfu; Figure 6A). The opposite effect, which was a relatively low electrostatic potential in the major groove of motif III, can be observed in the structure 433d (Trikha et al., 1999; Xu et al., 2007) (Figure 6C). The proximity of two asymmetrical tandem G•U pairs seemed to affect the helix geometry. As a result, the major groove was wider than the ones in a standard A-form RNA helix, and the pattern of the electrostatic potential was also altered.

**FIGURE 7:**
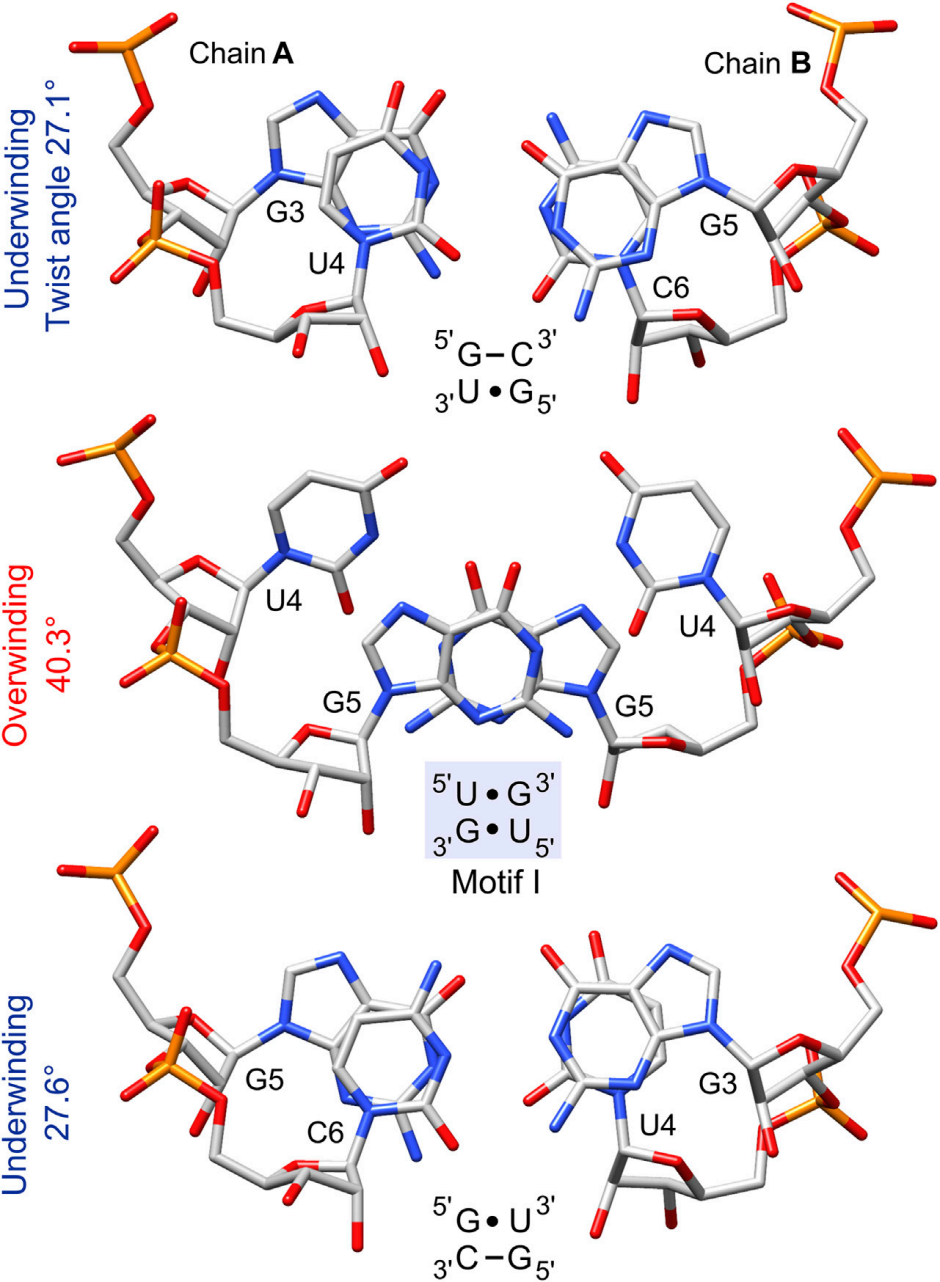
The stacking pattern within the 5′UG/3′GU tandem and between the wobble tandem pairs and flanking GC pairs. The 5′UG/3′GU wobble tandem represents motif I (*light blue box*), in which inter-strand stacking involves rings of guanosines, whereas unstacked uracils face each other. Local twist angle variations associated with each step are indicated.

The geometrical dissimilarity of the G•U pair with its U•G and WC counterparts (see below) affected the stacking interactions between the G•U pair and flanking base pairs (Ananth et al., 2013). The three G•U tandem motifs were characterized by different stacking patterns: I, purine (R)–R inter-strand stacking; II, R–pyrimidine (Y) intra-strand stacking; and III, a mix of intra- and inter-strand stacking (Figures 1 and 7). The reported diverse G•U tandem motifs in identical sequential contexts (PDB IDs 1eka and 1guc, 1qet and 1qes) showed varied stacking patterns with the flanking base pairs and differed in electrostatic potential (Figures 6A, B) (Xu et al., 2007). Moreover, the flanking sequences in 5′GAG-tandem-CUC3′ and in 5′GGA-tandem-UCC3′ seemed to exert opposite effects on the absolute value of the electrostatic potential for motif I (1eka, 1qet; Figure 6A) and motif II (1guc, 1qes; Figure 6B).

Overall, the sequential context of the wobble G•U pair and tandems impact the helix geometry, including the base stacking pattern and the major groove width. These structural features influence the overall electrostatic potential and eventual metal recognition and binding. It has been proposed that uniformity and the absence of positively charged groups make the major groove of G•U pairs an attractive binding site for metal ions to a greater extent than formal electrostatic potential (Chin et al., 1999). Colmenarejo and Tinoco observed that Mg^2+^ preferentially binds to G•U motif I, whereas complex ion [Co(NH_3_)_6_]^3+^ favorably binds to motifs II and III (1999). Thus, the binding affinity of specific RNAs is likely a composite of multiple factors involving geometry, electrostatic potential, hydration pattern, and some quantum effects (Xu et al., 2007). It was noted that Ba^2+^ is not a physiological cation and that the binding of Mg^2+^ occurs *in vivo* with two conceivable outcomes which may depend on the sequential context. One involves the direct coordination of Mg^2+^ by similar donors, as observed in our structure or in 1dfu, where Mg^2+^ recognition is affected by protein (Lu and Steitz, 2000). The second possibility is the indirect binding of hydrated Mg^2+^, only *via* water-mediated interactions. In both scenarios, the binding mode of Mg^2+^ could also change the RNA structure, and new structures with other flanking sequences are needed to elucidate more details. Knowledge about the predisposition of the G•U pair for binding cations is also useful in biochemical applications, for example, in crystallography to facilitate experimental phasing of X-ray structures (Cate and Doudna, 1996; Kieft and Tinoco, 1997; Keel et al., 2007).

### The 5′UG/3′GU Tandem in A-Helix Reveals Geometric Variations

All the calculations of the duplex structural parameters were performed using 3DNA v2.4.3 (2019apr06) (Li et al., 2019). For structural characterization of the 5′UG/3′GU tandem, unless specified otherwise, we have used simple base pairs and step parameters due to their more intuitive interpretation of structural variations in structures containing non-WC pairs. The simple parameters provide a complete qualitative description of the base-pairing geometries and their step parameters (Lu et al., 2015; Li et al., 2019). Hydrogen bonds in all WC pairs and the G•U wobble pairs of the presented duplex were in the expected range of 2.5–3.2 Å (Supplementary Table S2). The distance between the U4 (B) O2 atom and G5 (A) N2 atom in the wobble pair was 3.2 Å, indicating the presence of a weak hydrogen bond; however, the geometry was suboptimal for a stabilized interaction.

The global helix geometry resembled the A-form since the riboses revealed C3′-*endo* sugar puckers and the displacement from the helical axis (*X*-displacement) had a negative value, on average −4.46 Å, which is close to the ideal A-RNA helix, approx. −4 Å. However, a large negative *X*-displacement of −10.3 Å were observed at the 5′UG/3′GU step (local base-pair helical parameters; Supplementary Table S3). The individual torsion angles were within the ranges typical for the A-helix form (Table 2 and Supplementary Table S4) (Schneider et al., 1997). The average of the glycosidic dihedral angles (chi, χ) was −165 ± 3° for purines and was −163 ± 4° for pyrimidines. These values agree with the C3′-*endo* ribose and glycosidic bonds in *anti*-conformation (Table 2 and Supplementary Table S4). Similarly, the average of the delta (δ) angles of 82 ± 8° corresponded to the universal value for C3′-*endo* sugar conformation (81 ± 7°; Table 2 and Supplementary Table S4) (Gelbin et al., 1996; Parkinson et al., 1996; Schneider et al., 1997).

**TABLE 2:** Universal torsion angles for the A-helix and torsion angles (Gelbin et al., 1996; Parkinson et al., 1996; Schneider et al., 1997) in the r(UCGUGCGA)_2_ duplex calculated using the 3DNA server, v2.4.3-2019apr06 (Lu and Olson, 2003; Li et al., 2019).

Torsion angles (deg)				
	DNA A-form (Schneider et al., 1997)		*r*(UCGUGCGA)_2_	
Alpha (α)	−67 ± 17		−67 ± 8	
Beta (β)	174 ± 14		172 ± 5	
Gamma (γ)	56 ± 14		58 ± 8	
Delta (δ)	**C3**′**-*endo***		**C3**′**-*endo***	
	81 ± 7		82 ± 8	
Epsilon (ε)	−157 ± 12		−151 ± 5	
Zeta (ζ)	−71 ± 12		−71 ± 8	
Chi (χ) (Gelbin et al., 1996; Parkinson et al., 1996)	**C3**′**-*endo* purines**	**C3**′**-*endo* pyrimidines**	**C3**′**-*endo* purines**	**C3**′**-*endo* pyrimidines**
	−167 ± 14 [*anti*]	−164 ± 7 [*anti*]	−165 ± 3 [*anti*]	−163 ± 4 [*anti*]

The negative slide and positive roll values calculated for the present duplex (Table 3) are in agreement with the trend predicted for A-helix (Lu and Olson, 2003). Furthermore, the roll angles at each base-pair step alternated (Table 3). This tendency has been observed in alternating purine (R)–pyrimidine (Y) sequences in which the roll values tended to be lower at 5′R to 3′Y steps and higher at 5′Y–3′R steps (Dock-Bregeon et al., 1988; Dock-Bregeon et al., 1989; Biswas et al., 1997). The buckle angles tended to be negative for the 5′R–3′Y pairs and positive for the 5′Y–3′R pairs (Table 4).

**TABLE 3:** Simple base-pair step parameters of the r(UCGUGCGA)_2_ duplex based on consecutive C1′–C1′ vectors calculated using the 3DNA server, v2.4.3-2019apr06 (Lu and Olson, 2003; Li et al., 2019)

Base pair step	Shift (Å)	Slide (Å)	Rise (Å)	Tilt (deg)	Roll (deg)	Twist (deg)
5′UC/3′AG	−0.1	−1.6	3.3	1.5	3.9	30.1
5′CG/3′GC	−0.4	−1.6	3.3	−2.2	15.0	33.6
5′GU/3′CG	−0.1	−1.2	3.1	−0.3	9.1	27.1
5′UG/3′GU	1.0	−2.4	2.9	2.8	15.3	40.3
5′GC/3′UG	−0.4	−1.2	3.2	−1.7	5.3	27.6
5′CG/3′GC	0.1	−1.9	3.1	0.4	10.6	30.3
5′GA/3′CU	−0.2	−1.3	3.3	3.5	2.0	34.1
Average	0.0	−1.6	3.2	0.6	8.7	31.9
SD	0.5	0.4	0.1	2.2	5.3	4.6

**TABLE 4:** Simple base-pair parameters of the r(UCGUGCGA)_2_ duplex based on RC8-YC6 vectors calculated using the 3DNA server, v2.4.3-2019apr06 (Lu and Olson, 2003; Li et al., 2019)

Base pair	Shear (Å)	Stretch (Å)	Stagger (Å)	Buckle (deg)	Propeller (deg)	Opening (deg)
U–A	0.0	0.1	0.2	6.9	−9.8	3.9
C–G	0.6	−0.2	−0.1	6.6	−12.8	0.5
G–C	0.3	−0.1	0.0	−3.2	−15.2	1.7
U•G	2.5	−0.1	0.1	3.1	−11.0	−1.8
G•U	−2.3	−0.1	0.2	−5.2	−13.0	4.4
C–G	−0.1	−0.2	0.3	−1.4	−12.9	1.5
G–C	−0.4	−0.3	0.3	−3.6	−14.4	0.6
A–U	0.0	−0.4	0.0	−4.4	−13.2	0.1
Average	0.1	−0.2	0.1	−0.2	−12.8	1.4
SD	1.3	0.1	0.2	5.0	1.7	2.0

Several distinctive geometric features of the G•U wobble pair occurred in the 5′UG/3′GU tandem region. The key characteristic of the U•G and G•U wobble pairs is the shear of 2.5 and −2.4 Å, respectively (Lu and Olson, 2003) (Table 4 and Figure 3B). The displacement of the G•U pair relative to the WC counterparts provoked an asymmetry in the glycosidic bond angles (*λ*) subtended at the glycosyl carbon atoms C1 (Figure 3). The *λ* angles for the WC base pairs were nearly identical, measuring ∼54° (Figure 3A) (Varani and McClain, 2000). In wobble pairs, the *λ* angles measured ∼43° at G and ∼70° at U (Figure 3B and Supplementary Table S5). The above structural characteristic of the G•U pair caused its geometric dissimilarity (non-isostericity) with the WC base pairs and non-self-isostericity with the U•G pair (G•U ≠ U•G). The presence of the non-isosteric G•U contributes to the overall geometry of the RNA structures and may provide RNA recognition elements (Ananth et al., 2013).

The non-isostericity of G•U leads to the local twist angle variations (Ananth et al., 2013). The herein presented twist angle values around wobble pairs were calculated as simple base-pair step parameters in 3DNA based on the consecutive C1′–C1′ vectors (Li et al., 2019). Since the G•U tandem represents motif I, the 5′UG/3′GU tandem base-pair step had a high twist angle of 40.3°, while the steps between the wobble tandem and the flanking WC base pairs (5′GU/3′CG and 5′GC/3′UG) exhibited lower than average twist angles of 27.1° and 27.6° (Table 3 and Figure 7). The compensatory effect of the under- and overtwisting of the 5′UG/3′GU tandem and associated base pairs allowed the RNA duplex to maintain an average twist of ∼32°, which was close to the value for a typical A-RNA helix. A similar underwinding–overwinding–underwinding trend was also observed in other duplexes carrying the 5′UG/3′GU tandem motif I (e.g., PDB ID: 315d, 1eka) (Biswas et al., 1997; Chen et al., 2000).

The 5′UG/3′GU tandem was characterized by a stacking pattern typical of motif I (Figures 1, 7). At the U•G/G•U step, inter-strand stacking involved rings of guanosine, whereas unstacked uracils faced each other (Gautheret et al., 1995; Biswas et al., 1997) (Figures 1 and 7). The observed inter-strand stacking of G5 (A) and G5 (B) corresponded to overwinding (twist = 40.3°; Table 3 and Figure 7) and mostly resulted from the combination of a negative slide (−2.4 Å) and a positive shift (1 Å) (Lu and Olson, 2003) (Table 3). The strong stacking interaction between guanosines and the enhanced stacking of the U•G and G•U pairs with the flanking WC pairs (Figure 7) provided thermodynamic stability of the 5′UG/3′GU tandem—one of the major reasons making this motif the most abundant among G•U wobble tandem motifs (Gautheret et al., 1995; Wu et al., 1995; McDowell and Turner, 1996; Masquida and Westhof, 2000; Ananth et al., 2013). Comparison of the thermodynamic stability between the different wobble tandems revealed the following trend motif I > motif III > motif II (He et al., 1991; Wu et al., 1995; Deng and Sundaralingam, 2000). Moreover, the C–G pair succeeding G•U (as in the presented duplex) was thermodynamically more stable than G–C, A–U, and U–A (all after G•U) and, hence, was most frequent in the RNA structures (He et al., 1991; Ananth et al., 2013).

## Conclusion

G•U wobble base pairs play a key role in RNA biology. While several structures including such pairs have been reported, this work presents the first detailed analysis based on an experimental model of the motif I G•U tandem complexed with a divalent metal cation. This is particularly important as metal binding by G•U pairs appears vital to RNA folding, catalytic functions, and RNA–protein interactions.

The metal binding site in the r(UCGUGCGA)_2_ duplex was formed by the G•U pair tandem in the major groove. The O6 carbonyl of G and two O4 carbonyls of U within the tandem created the coordination sphere of the metal. The G•U pairs were non-self-isosteric (G•U ≠ U•G), which is consistent with the asymmetric coordination sphere of the metal cation. It is also apparent that the distribution of the electrostatic potential within the major groove contributed to the formation of the metal binding site. The aforementioned features may provide elements for recognition by RNA binding proteins.

## Materials and Methods

### Crystallization Conditions and Diffraction Data Collection

The crystal of the r(UCGUGCGA)_2_ duplex was obtained in the presence of its complementary strand UCGCACGA. The crystals grew in the 12th condition of nucleic acid mini-screen from Hampton [10% *m*-phenylenediamine (MPD), 0.04 M sodium cacodylate trihydrate, pH 6.0, 0.012 M spermine tetrahydrochloride, 0.08 M potassium chloride, and 0.02 M barium chloride] in 2 weeks with size of ∼0.1 × 0.06 × 0.03 mm, using a hanging drop setting. They were cryoprotected by increasing the MPD concentration to 35%.

Diffraction data were collected at the SER-CAT beamline 22-BM at the Advanced Photon Source, Argonne National Laboratory (Lemont, IL, USA). The diffraction data were processed with the XDS package (Kabsch, 2010); the processing statistics are given in Table 1.

### Determination and Refinement of the Crystal Structures

The crystal structure of the r(UCGUGCGA)_2_ duplex was solved by molecular replacement in PHASER (McCoy et al., 2007) using an ideal single-stranded RNA, generated in *Coot* (Emsley et al., 2010). The data were also processed in the *H*3 space group and tested for twinning using *L*-test and by twin refinement in Refmac5 (Murshudov et al., 2011). The estimated twin fraction was ∼20%, and *R*_work_/*R*_free_ did not decrease; therefore, we decided to use data in the *H*32 space group. The initial model was refined initially in phenix.refine (Afonine et al., 2012) and in the final cycles in Refmac5 (Murshudov et al., 2011) with external restraints generated by the RestraintLib (Kowiel et al., 2020) server. *Coot* (Emsley et al., 2010) was used for manual model corrections between rounds of automatic model refinement. The presence of Ba^2+^ was inferred from the peak size of the electron density map, correlated with the content of the crystallization solution. The anomalous difference electron density map, calculated with Phenix (Liebschner et al., 2019), confirmed the presence of an anomalous scatterer, interpreted as Ba^2+^.

### Determination of Structural Parameters and Electrostatic Potential

Structural parameters of the r(UCGUGCGA)_2_ duplex were analyzed using the 3DNA (v2.4.3-2019apr06) server (Li et al., 2019). The electrostatic potential of the r(UCGUGCGA)_2_ duplex and the other structures compared in this study [PDB ID: 1eka, 1qet, 1dfu, 1qes, 1guc, 1c0o, 433d, 472d, and 1ajf; ideal A-helix r(UCGCGCGA)_2_ generated by *COOT*] (Emsley et al., 2010) was calculated using the APBS/PDB2PQR server (Baker et al., 2001; Dolinsky et al., 2004; Jurrus et al., 2018) with default settings (accessed in March 2021). The results of the electrostatic potential calculations were presented using *Chimera 1.13.1* software (Pettersen et al., 2004).

## Data Availability

Atomic coordinates and structure factors for the crystal structure of RNA duplex r(UCGUGCGA)_2_ in complex with Ba^2+^ cation have been deposited with the Protein Data Bank (PDB) under the PDB ID 7ouo.
