# Supplementary material for: Structural Insights Into the 5′UG/3′GU Wobble Tandem in Complex With Ba2+ Cation
Source: Front Mol Biosci. 2022 Jan 13;8:762786. doi: 10.3389/fmolb.2021.762786 (PMC8793689; doi:10.3389/fmolb.2021.762786)
Supplement: Supplementary file 1 [file DataSheet1.pdf]

SUPPLEMENTARY DATA

Structural insights into the 5'UG/3'GU wobble tandem in complex with Ba^{2+} cation

Agnieszka Ruszkowska[1], Ya Ying Zheng[2], Song Mao[2], Milosz Ruszkowski[1*], Jia Sheng[2*]

[1]Institute of Bioorganic Chemistry, Polish Academy of Sciences, 61-704 Poznan, Poland.

[2]Department of Chemistry, and The RNA Institute, University at Albany, State University of New York, Albany, NY, USA. jsheng@albany.edu

Table S1. Minor and major grooves dimensions in ideal RNA A-helix (generated by *COOT* software (Emsley et al., 2010)) and the r(UCGUGCGA)^2 duplex. Calculations were performed by 3DNA server (Lu and Olson, 2003; Li et al., 2019) v2.4.3-2019apr06.

**r(UCGUGCGA)$_2$ duplex**					**Ideal RNA A-helix**				
	**Minor Groove**		**Major Groove**			**Minor Groove**		**Major Groove**	
step	P-P (Å)	Refined[A] (Å)	P-P (Å)	Refined[A] (Å)	step	P-P (Å)	Refined[A] (Å)	P-P (Å)	Refined[A] (Å)
**5'GU/3'CG**	17.5	---	15.6	---	**5'GU/3'CG**	18.7	---	14.9	---
**5'UG/3'UG**	17.4	15.7	16.5	10.7	**5'CG/3'CG**	18.7	16.8	14.9	12.1
**5'GC/3'UG**	16.8	---	17.4	---	**5'GC/3'UG**	18.7	---	14.9	---

[A]refined P-P distances take into account the directions of the sugar-phosphate backbones.

Table S2. Hydrogen-bonding interactions within the r(UCGUGCGA)^2 duplex calculated by 3DNA server (Lu and Olson, 2003; Li et al., 2019) v2.4.3-2019apr06.

Pair nr	Base Pair	H-bonds	Atoms	Distance (Å)
1	**U-A**	[2]	N3 - N1	3.0
			O4 - N6	3.2
2	**C-G**	[3]	N4 - O6	3.0
			N3 - N1	2.9
			O2 - N2	2.8
3	**G-C**	[3]	O6 - N4	3.0
			N1 - N3	2.9
			N2 - O2	2.7
4	**U•G**	[2]	N3 - O6	2.7
			O2 - N1	2.7
5	**G•U**	[2]	O6 - N3	2.8
			N1 - O2	2.5
6	**C-G**	[3]	N4 - O6	2.9
			N3 - N1	2.8
			O2 - N2	2.6
7	**G-C**	[3]	O6 - N4	2.9
			N1 - N3	2.8
			N2 - O2	2.7
8	**A-U**	[2]	N1 - N3	2.6
			N6 - O4	2.7

Table S3. Local base-pair helical parameters of the r(UCGUGCGA)^2 duplex calculated by 3DNA server(Lu and Olson, 2003; Li et al., 2019) v2.4.3-2019apr06.

**step**	**X displacement (Å)**	**Y displacement (Å)**	**Inclination (°)**	**Tip (°)**
**5'UC/3'AG**	-3.3	0.4	6.6	-2.7
**5'CG/3'GC**	-4.8	0.6	25.9	2.6
**5'GU/3'CG**	-2.7	0.5	13.5	-1.3
**5'UG/3'GU**	-10.3	-2.0	46.3	-8.7
**5'GC/3'UG**	-2.4	0.1	7.4	3.3
**5'CG/3'GC**	-5.5	-0.3	20.7	-0.2
**5'GA/3'CU**	-2.3	0.8	3.3	-5.5
**ave.**	-4.5	0.0	17.7	-1.8
**s.d.**	2.9	0.9	15.0	4.3

Table S4. Torsions angles in the r(UCGUGCGA)[2] duplex calculated by 3DNA server(Lu and Olson, 2003; Li et al., 2019) v2.4.3-2019apr06.

		Torsion angles (°)						
base		Alpha (α)	Beta (β)	Gamma (γ)	Delta (δ)	Epsilon (ϵ)	Zeta (ζ)	Chi (χ)
				Chain A				
1	U	---	---	51.5	83	-144.5	-76	-161.1
2	C	-60.4	166.8	59.8	78.5	-150.1	-79.6	-161.8
3	G	-62.1	174.4	50.9	79.9	-150.1	-57.8	-166.6
4	U	-77.3	170.8	58.8	82	-149.8	-75.8	-160.1
5	G	-64.6	161.5	64.2	75.6	-152.1	-66	-166.5
6	C	-80.1	175.3	60.8	74	-153.6	-65.7	-171.2
7	G	-69.7	176.8	64.1	78.3	-149.2	-78	-167.8
8	A	-61.7	166.8	70.6	105.6	---	---	-160.4
				Chain B				
1	A	-77.9	179.6	71.3	92.1	---	---	-162.8
2	G	-70.8	173.4	59.9	78.6	-162.1	-61	-169
3	C	-73.6	171.4	58.3	77	-147.1	-72	-164.9
4	G	-57	176.3	42.2	77.8	-151.1	-63.7	-162.9
5	U	-60.2	166.2	53.9	80.5	-160.2	-86.7	-158.7
6	G	-64	175	50.9	78.5	-148.2	-70.5	-166.2
7	C	-59.8	167.6	50.4	80.7	-147.7	-72.9	-164.1
8	U	---	---	62.2	83.6	-142.2	-72.8	-164.2

Table S5. The glycosidic bond angles (lambda, λ) observed in the r(UCGUGCGA)[2] duplex. Calculations were performed by 3DNA server (Lu and Olson, 2003; Li et al., 2019) v2.4.3-2019apr06. Values characteristic for G•U pair are marked by red and described in the main text.

Base pair	lambda (λ,°)	
**U-A**	57.5	57.6
**C-G**	58.7	55.9
**G-C**	55.6	53.6
**U•G**	69	42.2
**G•U**	44.3	70.3
**C-G**	56.3	58.6
**G-C**	56	58.2
**A-U**	54.9	53.5

References

Emsley, P., Lohkamp, B., Scott, W.G., and Cowtan, K. (2010). Features and development of Coot. *Acta Crystallogr D Biol Crystallogr* 66**,** 486-501. doi: 10.1107/S0907444910007493

Li, S., Olson, W.K., and Lu, X.J. (2019). Web 3DNA 2.0 for the analysis, visualization, and modeling of 3D nucleic acid structures. *Nucleic Acids Res* 47**,** W26-W34. doi: 10.1093/nar/gkz394

Lu, X.J., and Olson, W.K. (2003). 3DNA: a software package for the analysis, rebuilding and visualization of three-dimensional nucleic acid structures. *Nucleic Acids Res* 31**,** 5108-5121. doi: 10.1093/nar/gkg680
